# Eye-Tracking Feature Extraction for Biometric Machine Learning

**DOI:** 10.3389/fnbot.2021.796895

**Published:** 2022-02-01

**Authors:** Jia Zheng Lim, James Mountstephens, Jason Teo

**Affiliations:** ^1^Evolutionary Computing Laboratory, Faculty of Computing and Informatics, Universiti Malaysia Sabah, Kota Kinabalu, Malaysia; ^2^Faculty of Computing and Informatics, Universiti Malaysia Sabah, Kota Kinabalu, Malaysia

**Keywords:** classification, eye-tracking, fixation, biometric machine learning, feature extraction

## Abstract

**Context:**

Eye tracking is a technology to measure and determine the eye movements and eye positions of an individual. The eye data can be collected and recorded using an eye tracker. Eye-tracking data offer unprecedented insights into human actions and environments, digitizing how people communicate with computers, and providing novel opportunities to conduct passive biometric-based classification such as emotion prediction. The objective of this article is to review what specific machine learning features can be obtained from eye-tracking data for the classification task.

**Methods:**

We performed a systematic literature review (SLR) covering the eye-tracking studies in classification published from 2016 to the present. In the search process, we used four independent electronic databases which were the IEEE Xplore, the ACM Digital Library, and the ScienceDirect repositories as well as the Google Scholar. The selection process was performed by using the Preferred Reporting Items for Systematic Reviews and Meta-Analyses (PRISMA) search strategy. We followed the processes indicated in the PRISMA to choose the appropriate relevant articles.

**Results:**

Out of the initial 420 articles that were returned from our initial search query, 37 articles were finally identified and used in the qualitative synthesis, which were deemed to be directly relevant to our research question based on our methodology.

**Conclusion:**

The features that could be extracted from eye-tracking data included pupil size, saccade, fixations, velocity, blink, pupil position, electrooculogram (EOG), and gaze point. Fixation was the most commonly used feature among the studies found.

## Introduction

There is now an increasing interest in the interplay between artificial intelligence (AI) and human–computer interaction (HCI). Scientific researchers are now putting increasingly significant efforts into investigating novel interactions between humans and machines. The studies on classification using machine learning have become very popular including emotion prediction as well as image classification, since it can learn automatically and perform specific tasks at human-level capabilities without the intervention of a human expert. Therefore, there are studies on classification using different approaches such as emotion classification using brainwave signals (Ullah et al., 2019) and image classification using neural networks (Yang et al., 2016). While a representative study on learning machines, the study of Nilsson (1965) was more concerned with machine learning for pattern classification. The studies on eye tracking also increased in recent years. Many researchers conduct their experiments with the question of how eye-tracking data can be utilized in their research. Hence, the usage of eye-tracking technology in classification research springs up with the doubt of what eye features can be obtained from eye-tracking data for classification.

Eye-tracking technology refers to the process of tracking and measuring the eye movements and the focus point of the eyes of the user. Eye tracking is widely used in many domains such as psychology, marketing, medical, computer gaming, and cognitive science. Therefore, eye tracking is increasingly used in computer science fields and utilizes eye features to study information processing tasks (Rayner, 2009). Eye-tracking data can be measured and obtained by using an eye-tracking sensor or a camera. The data provide several features, and it can be used for several classification tasks. Eye-tracking technology is very helpful and it can be widely adopted and implemented in the future, as it only requires a simple camera to collect the data needed.

In this article, we present a systematic literature review and collect all the studies and articles relevant to the usage of features that can be obtained from eye-tracking data for classification within 5 years, i.e., from 2016 to the present. The first section presents the introduction of this article. In Background section, we provide a background on eye-tracking technology and eye-tracker types including desktop eye-tracking, mobile eye-tracking, and eye tracking in virtual reality (VR) as well as a brief introduction of machine learning. A methodology of research is described in Methodology section that included research question, selection criteria, search process, and selection process. Results section presents the results and the related studies are shown in Table 1. The final section concludes this article.

**Table 1:** Summary of studies using eye features.

**References**	**Year**	**Topic domain**	**Objective**	**Eye features**	**Subjects**	**Eye-trackers**	**Classifier**	**Performance**
Cao et al. (2016)	2016	Intention recognition	To examine and evaluate whether pupil variation has a relevant impact on the endoscopic manipulator activation judgment	Pupil size, velocity of eye rotation	12 (10 males, 2 females)	Tobii 1750	SVM and PNN	88.6%
Ahmed and Noble (2016)	2016	Image classification	Attempt to classify and acquiring the image frames of the head, abdominal, and femoral from 2-D B-Mode ultrasound scanning	Fixations	10	EyeTribe (30Hz)	Bag of words model	85–89%
Zhang and Juhola (2016)	2017	Biometric identification	To study primarily biometric recognition as a multi-class classification process and biometric authentication as binary classification	Saccades	109	EyeLink (SR Research)	SVM, LDA, RBF, MLP	80–90%
Zhou et al. (2017)	2017	Image classification	To propose an approach of two-stage feature selection for image classification by considering human factors and leveraging the importance of the eye-tracking data.	Fixations, ROI	-	Tobii X120	SVM	94.21%
Borys et al. (2017)	2017	User performance classification in RFFT	To verify and evaluate whether eye-tracking data in combination with machine learning could be used to identify user output in RFFT.	Fixations, saccades, blinks, pupil size	61	Tobii Pro TX300	Quadratic discriminant analysis	78.7%
Karessli et al. (2017)	2017	Image classification	To propose an approach that uses gaze data for zero-shot image classification	Gaze point	5	Tobii TX300 (300Hz)	SVM	78.2%
Labibah et al. (2018)	2018	Lie detection	To construct the object using a lie detector with the analysis of pupil changes and eye movements using image processing and decision tree algorithm.	Pupil diameter, eye movements	40	Computer camera	Decision tree	95%
Qi et al. (2018)	2018	Material classification	To investigate how humans interpret material images and find information on eye fixation enhances the efficiency of material recognition.	Fixation points, gaze paths	8	Eye-tracker	CNN	85.9%
Singh et al. (2018)	2018	Reading pattern classification	To analyze the reading patterns of eye-tracking inspectors and assesses their ability to detect specific types of faults.	Fixations, saccades	39	EyeLink 1000	NB, MNB, RF, SGD, ensemble, decision trees, Lazy network	79.3–94%
Lagodzinski et al. (2018)	2018	Cognitive activity recognition	To discuss the concept of the eye movement study, which can be used effectively in behavior detection due to the good connection with cognitive activities.	EOG, accelerometer data	100	JINS MEME EOG-based eye-tracker	SVM	99.3%
Bozkir et al. (2019)	2019	Cognitive load classification	To propose a scheme for the detection of cognitive driver loads in safety-critical circumstances using eye data in VR.	Pupil diameter	16	Pupil Labs	SVM, KNN, RF, decision trees	80%
Orlosky et al. (2019)	2019	User understanding recognition	To recognize the understanding of the vocabulary of a user in AR/VR learning interfaces using eye-tracking.	Pupil size	16	Pupil Labs Dev IR camera	SVM	62–75%
Sargezeh et al. (2019)	2019	Gender classification	To examine parameters of eye movement to explore gender eye patterns difference while viewing the indoor image and classify them into two subgroups.	Saccade amplitude, number of saccades, fixation duration, spatial density, scan path, RFDSD	45 (25 males, 20 females)	EyeLink 1000 plus	SVM	84.4%
Tamuly et al. (2019)	2019	Image classification	To develop a system for classifying images into three categories from extracted eye features.	Fixation count, fixation duration average, fixation frequency, saccade count, saccade frequency, saccade duration total, saccade velocity total	25	SMI eye-tracker	KNN, NB, decision trees	57.6%
Luo et al. (2019)	2019	Object detection	To develop a framework for extracting high-level eye features from low-cost remote eye-tracker's outputs with which the object can be detected.	Fixation length, radius of fixation, number of time-adjacent clusters	15 (6 males, 9 females)	Tobii Eye Tracker 4C	SVM	97.85%
Startsev and Dorr (2019)	2019	ASD classification	To propose a framework that identifies an individual's viewing activity as likely to be correlated with either ASD or normal development in a fully automated fashion, based on scan path and analytically expected salience.	Fixations, scan path	14	Tobii T120	RF	76.9% AUC
Zhu et al. (2019)	2019	Depression recognition	To propose a depression detection using CBEM and compare the accuracy with the traditional classifier.	Fixation, saccade, pupil size, dwell time	36	EyeLink 1000	CBEM	82.5%
Vidyapu et al. (2019)	2019	Attention prediction	To present an approach for user attention prediction on webpage images.	Fixations	42 (21 males, 21 females)	Computer webcam	SVM	67.49%
Kacur et al. (2019)	2019	Schizophrenia disorder detection	To present a method to detect schizophrenia disorder using the Rorschach Inkblot Test and eye-tracking.	Gaze position	44	Tobii X2-60	KNN	62% - 75%
Yoo et al. (2019)	2019	Gaze-writing classification	To propose a gaze-writing entry method to identify numeric gaze-writing as a hands-free environment.	Gaze position	10	Tobii Pro X2-30	CNN	99.21%
Roy et al. (2017)	2020	Image identification	To develop a cognitive model for ambiguous image identification.	Eye fixations, fixation duration, pupil diameter, polar moments, moments of inertia	24 (all males)	Tobii Pro X2-30	LDA, QDA, SVM, KNN, decision trees, bagged tree	~90%
Guo et al. (2021)	2021	Workload estimation	To investigate the usage of eye-tracking technology for workload estimation and performance evaluation in space teleoperation	Eye fixation, eye saccade, blink, gaze, and pupillary response	10 (8 males, 2 females)	Pupil Labs Core	LOSO protocol, SVM (RBF)	49.32%
Saab et al. (2021)	2021	Image classification	To propose an observational supervision approach for medical image classification using gaze features and deep learning	Gaze data	-	Tobii Pro Nano	CNN	84.5%

## Background

### Eye-Tracking Technology

Eye-tracking technology is an emerging technology used to monitor the eye movements of a user or the focus point of an individual. It is a process of measuring the point of gaze or the position of eyes and collecting the eye features from an individual and it is recorded in the form of data, which is comprehensive statistics such as fixation counts, first fixation, and fixation duration. These recorded data can be analyzed by using visual analytic approaches to study and extract the eye features. Applying a visual analytic technique is to improve visualization of common visual problem-solving strategies (Andrienko et al., 2012). Eye data also can be explored and analyzed graphically using data visualization software such as heatmaps and saliency maps.

Eye-tracking data can be categorized on the basis of the essential ocular activity indicators, which are fixations, saccades, and scan path. Fixations are eye motions stabilizing the retina above a stationary object of interest with a duration of 100–400 ms. The fixations are fairly centered and the eye travels at low velocity. They are characterized by ocular drifts, ocular microtremor, and microsaccades (Pritchard, 1961). Saccades are quick movements of both eyes used to reposition the fovea, the middle part of the retina, into a new location in the visual environment. Saccadic movements are typically in duration from 10 to 100 ms and they are reflexive and voluntary (Duchowski, 2017). There are four types of saccade classifications, which are predictive saccade, antisaccade, memory-guided saccade, and visually-guided saccade (Rommelse et al., 2008). Scan path of the eye movement is defined as the direction taken by the eyes of the viewer when reading a text or observing a scene. The scan path data is the information of the trajectories of the eyes as the visual field is scanned and some kind of visual information is interpreted and analyzed. A scan path is the resulting series of saccades and fixations.

### Eye Tracker

An eye tracker is a device to detect eye movements and eye positions. It is built to measure the visual attention of a subject by gathering data on eye movement when the subject observes a stimulus while operating on a task. There are three types of eye tracker, which are eye-attached tracking, optical tracking, and measurement of electric potentials with electrodes. Eye-attached tracking is a measurement by using an eye attachment, for example, a special contact lens. The movement of the attachment is calculated on the basis that it does not shift dramatically as the eye rotates. This method allows for measuring the movement of the eyes in horizontal, vertical, and torsional directions (Robinson, 1963). Optical tracking determines the position of an object in real-time by monitoring the positions that are attached to the object. The location of the reflex point is determined by means of a camera device. The optical method tracks eye movement without direct contact with the eye. This method is commonly used for gaze tracking, especially those based on video capture, and is preferred for being affordable and non-invasive. The third type of eye tracker is the measurement of electric potentials with electrodes. The eyes are the source of a permanent electrical field that can be observed in complete darkness even when the eyes are closed. An example of this tracking method is the electrooculogram (EOG). It is a technique used to measure the corneo-retinal standing potential that occurs between the forehead and back of the human eye. It is a very lightweight solution that needs only very low computing power. It also performs under various lighting conditions and it can also be implemented as an integrated, self-contained wearable device (Sopic et al., 2018).

### Machine Learning

Machine learning is an AI computer algorithm that automatically develops with practice. It creates a model based on the training data to make predictions without being specifically programmed to do so (Koza et al., 1996). In cognitive science, the performance of emotion classification using machine learning is validated by precision or accuracy estimation techniques. The most commonly used machine learning algorithms included support vector machine (SVM), K-nearest neighbor (KNN), and random forest. The predictions and classification tasks are done based on the features from datasets (Kootstra et al., 2020). Machine learning can be applied to many fields such as computer vision, where it is unworkable to create traditional algorithms to perform the tasks required. A scientific report proposed an approach to classifying the age of toddlers based on gaze patterns (Dalrymple et al., 2019). There is also a study on detecting personality traits using eye tracking from external stimuli (Berkovsky et al., 2019). Machine learning also involves computer learning from information or data given, so that certain tasks are performed.

## Methodology

### Research Question

What features can be obtained from eye-tracking data for classification?

### Selection Criteria

The selection included the studies on classification using eye-tracking technology, which focuses on what features are used from the eye-tracking data for the classification tasks. The term “classification” refers to all the categorization activities such as cognitive states, intentions, actions, or events. All the features used or extracted from eye-tracking data are included in the selection criteria.

### Search Process

In the search process, we used independent electronic databases such as IEEE Xplore,^1^ ACM Digital Library,^2^ and ScienceDirect^3^ digital libraries as well as Google Scholar^4^ separately. We ensure that the search query is the same for every digital library. We search the studies including the research articles, journals, and conferences within 6 years (2016 to present) with a unique search query. According to the research question, the searching query is “eye tracking for classification.” Since our objective is to investigate what eye features can be obtained from eye-tracking data in classification, hence eye tracking and classification are used as our keywords in the searching query. Therefore, we presented the two sets of keywords with a quotation mark, which is between a “ ”. We collected all the articles and a reference management software, called Mendeley^5^ is used to save and manage the articles.

### Selection Process

We select the appropriate-related articles from the 4 digital libraries and ensure that there is no duplicated article in the selection process. The selection is executed with the Preferred Reporting Items for Systematic Reviews and Meta-Analyses (PRISMA) guidelines and the articles are managed in Mendeley. In Mendeley, we applied the tools to be checked for duplicates to remove the same articles. We remove all the unrelated articles by searching the keywords in articles including the titles, abstracts, and contents. Full-text articles are retrieved for final inclusion. Since our research is related to eye tracking, hence the keywords are “eye tracking,” “eye tracker,” “eye,” “gaze,” and “pupil.” Those articles that have no results from these searching keywords are removed in the eligibility process. The articles that have <20 from the searching keywords are checked by reading the methodology of the article to ensure that eye tracking is used in the investigation. In the last process, we choose the studies in qualitative synthesis to ensure that the article is matched to our research question. Those articles that use eye tracking, but not for classification tasks are also removed.

## Results

**Figure 1:**
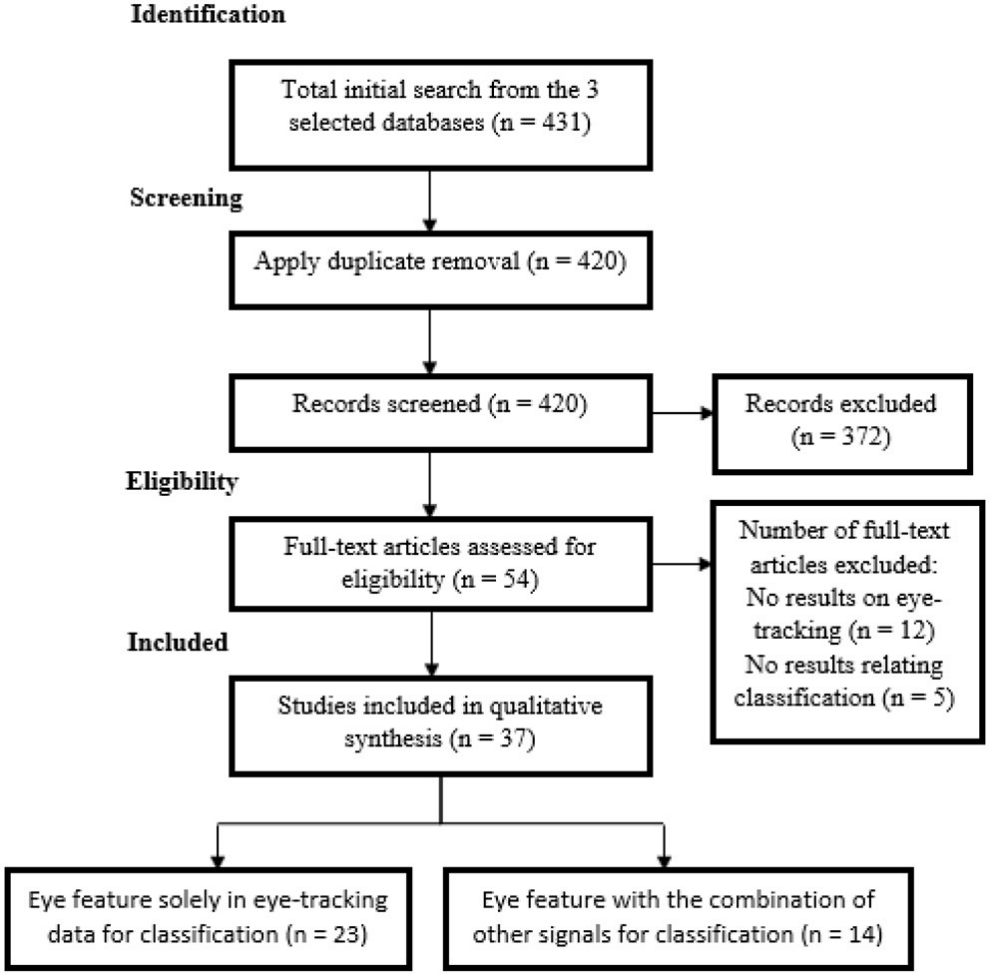
Selection process with the Preferred Reporting Items for Systematic Reviews and Meta-Analyses (PRISMA) search strategy.

Initial results returned a total number of 431, which are 33 from the ScienceDirect digital library, 92 from the IEEE Xplore digital library, and 306 from the Association for Computing Machinery (ACM) digital library. These articles were also available from the Google Scholar search engine. Through the screening process, there were 420 articles remaining after applying duplicate removal in Mendeley. After preliminary screening of these 420 articles, there were 54 full-text articles found to be relevant from the search results. However, there were finally only 37 studies that were included in the qualitative synthesis as there were 12 articles that did not have results relating to eye tracking and 5 articles that did not have results relating to classification. The studies were divided into 2 categories, which are the 23 studies that use eye features solely from eye-tracking data for classification and 14 studies that use eye features with a combination of other signals for classification. Figure 1 shows the selection process with the PRISMA strategy.

### Pupil Size

Cao et al. (2016) proposed an approach for the intention recognition system with an endoscopic manipulator using pupil size and velocity of eye rotation. The study showed that pupil variation has a significant impact on the control of an endoscope. In a study by Labibah et al. (2018), the authors constructed an experiment to determine someone is lying or not using the changes of pupil diameter and the movement of the eyeball, which is left or right. The determination of lies is classified using a decision tree algorithm. Eye-tracking technology is also widely used in security. In a study by Bozkir et al. (2019), a gaze-driven cognitive load recognition scheme for drivers in safety-critical circumstances is proposed based on pupil diameter in VR. In a study by Orlosky et al. (2019), the authors conducted an experiment to recognize the understanding of vocabulary and language of a user in augmented reality (AR) and VR interfaces.

In a study by Slanzi et al. (2017), the authors assessed an experiment to predict the click intention of a user when browsing a website using the combination of pupil dilation and electroencephalogram (EEG) signals. The studies found that it has a greater pupil size when a user is willing to click. In another study by Guo et al. (2019), a multimodal emotion recognition investigation is proposed using the three modalities, which are eye movements such as pupil diameter and blink, EEG, and eye images (EIG).

### Saccade

In a study by Zhang and Juhola (2016), the investigators studied biometric identification and verification using saccadic eye movement signals. The performances are good with the highest identification rate of 90%. In another study by Sargezeh et al. (2019), the authors investigated the eye movement parameters such as saccade amplitude, number of saccades, and the ratio of total fixation duration to total saccade duration (RFDSD) to classify the genders. In a study by Tamuly et al. (2019), an image classification investigation is proposed to predict the valence of the scene using multiple features of eye movements such as saccade count and saccade frequency, along with machine learning.

### Fixation

Eye movement data is also widely used in medical and clinical analysis. From the study of Ahmed and Noble (2016), the authors performed a fetal ultrasound (US) image classification for obtaining the standardized abdominal, head, and femoral image frames using extracted eye fixations. The findings showed that producing a bag of models of words using fixations is a promising method for identifying fetal US images. Zhou et al. (2017) presented an investigation on image classification using a method of two-stage feature selection. The feature is based on the region of interest (ROI), which is identified from the extracted fixation data. Qi et al. (2018) utilized fixation points and gaze paths for material recognition and improve the performance by generating saliency maps. The study of Singh et al. (2018) analyzes the reading patterns using fixations and saccades with multiple classifiers to determine which are the best for the evaluation. In another study by Luo et al. (2019), a gaze-based approach is proposed for the intention detection using fixation duration and radius of fixation. In a study by Startsev and Dorr (2019), the authors classified autism spectrum disorder (ASD) based on scan path and saliency features. There is a study by Zhu et al. (2019) in which they conducted two separate emotional experiments in the investigation based on EEG and eye-tracking data solely with a content-based ensemble method (CBEM) as a classifier. Fixations capture the visual attention of an individual when focusing on an interesting thing. Vidyapu et al. (2019) proposed an attention prediction on webpage images using multilabel classification. In a study by Roy et al. (2017), the authors developed a method for the cognitive process to identify ambiguous images.

Furthermore, there are 3 articles that used the combination of EEG with fixations in the investigations. In a study by Shi et al. (2017), an attention evaluation method is proposed with the combination of EEG, fixations, and scan path for the automated classification of high- and low-quality data using a spatial-temporal scan path analysis. According to a study by Czyzewski et al. (2017), they proposed a multimodal approach for polysensory treatment and stimulation of noncommunicative subjects and classified the real and imaginary motion of limbs using gaze fixation points and EEG. In a study by Jiang et al. (2019), a study on the classification of ASD is proposed using face features and eye fixations, while in a study by Thapaliya et al. (2018), the authors utilized the combination of fixation times and EEG to classify the diagnosis of ASD. The studies showed that it has a better performance with combined data comparing to EEG or eye data solely. Besides that, there is a study by Ding et al. (2019) on the detection of major depressive disorder (MDD) using three combinations of data, which are eye-tracking data, EEG, and galvanic skin responses (GSRs) data. Furthermore, there is a study by Abdelrahman et al. (2019) that proposed a method for the classification of attention types by using fixation data and thermal imaging.

### Velocity

From the study of Koza et al. (1996), the authors proposed an endoscopic manipulator purpose recognition program based on 2 classifiers, which are support vector machine (SVM) and probabilistic neural network (PNN). The feature of pupil variation and the velocity of eye rotation are used in the investigation. The studies found that pupil variation has a significant influence on the timing of activation of the endoscopic manipulator to move the operating field to the middle of the visual field of the monitor.

### Blink

In a study by Borys et al. (2017), the authors conducted an experiment on user performance classification based on the Ruff Figural Fluency Test (RFFT) using machine learning and eye features such as blinks, pupil size, fixations, and saccades. The RFFT assesses the ability of an individual to generate new figures using five different dot configurations. In a study by Guo et al. (2021), the authors conducted an experiment for workload estimation and explore the usage of eye-tracking technology in performance evaluation in space teleoperation with two confounding factors, which are latency and time pressure. The eye features used in this investigation included eye blink, fixation, saccade, gaze, and pupil diameter. The workload recognition performance is evaluated using the proposed method, a leave-one-subject-out (LOCO) protocol. Four-class workload recognition is done and the best accuracy obtained was 49.32%.

In a study by Guo et al. (2019), the authors utilized blink and pupil diameter for five-class emotion recognition with the combination of EEG and EIG. In a study by Ha et al. (2021), the authors created a meal assistant device to improve the self-esteem and enhance the life quality of disabled and elderly people. The proposed brain–computer interface (BCI) system is developed based on the features of triple eye blinks, EEG, and EMG. This study showed positive results with an accuracy range of 83–97%.

### Pupil Position

Recently, eye-tracking technology is emerging in the detection of diagnosis of medical purposes. In a study by Kacur et al. (2019), the authors presented a method for the detection of schizophrenia disorders using gaze position with the Rorschach Inkblot Test. In a study by Yoo et al. (2019), a gaze-writing classification technique for numeric gaze-written entry is proposed.

There is research that used the combination of pupil position and functional MRI (fMRI) signals for the decoding of bistable plaid motion perception (Wilbertz et al., 2018). Bistable perception describes a condition in which conscious perception continuously alternates between two potential perceptions of a physically consistent but perceptibly ambiguous stimulus. In a study by Lin et al. (2019), a mental spelling system is proposed using the combination of gaze position and steady-state visual evoked potentials (SSVEPs) features from brainwave with filter bank canonical correlation analysis (FBCCA) for classification.

### Electrooculography

Electrooculography is a method used to calculate the corneo-retinal standing potential between the front eye and back eye of the human. In a study by Lagodzinski et al. (2018), the authors conducted an experiment on cognitive activity recognition using EOG signals with a machine learning algorithm, codebook approach.

In a recent study by Kubacki (2021), the author proposed a novel hybrid BCI system for item sorting. The research is carried out using an industrial robot in the virtual model. The controlling of the robot is done by using the features of SSVEP, EOG, eye position, and force feedback. On a real-world industrial robot, the tests are replicated. The verification of the positioning accuracy of the robot tip is done with the feedback system. This research found that the proposed system can sort items using the signals of the human body and maintained the accuracy rate at 90%. The study by Song et al. (2021) proposed a deep coupling recurrent auto-encoder (DCRA) for vigilance estimation using the combination of EEG and EOG. In this study, the design of the auto-encoder is done by using the gated recurrent units (GRUs). The EEG and EOG data were recorded using a neuroscan system, while eye movement data were collected using eye-tracking glasses, which included eye blinking, closing eyes, fixation, and saccade. The level of driver alertness SEED-VIG (Zheng and Lu, 2017) is used as the dataset for the simulation experiment.

### Gaze Point

There is only a study that used gaze point as the eye feature from eye-tracking data. In a study by Horng and Lin (2019), the authors designed an experiment on drowsiness prediction and classification using multimodal biosignals such as eye movements, GSR, brainwave signals, and heart rate. In a study by Karessli et al. (2017), the authors utilized the gaze attributes and embeddings such as gaze features with grid (GFG), gaze features with sequence (GFS), and gaze histogram (GH) for zero-shot image classification. The extracted gaze data included gaze points, gaze location, gaze duration, gaze sequence as well as pupil diameter. The classifier used in this investigation was SVM and the highest accuracy obtained was 78.2%. In a study by Saab et al. (2021), the authors collected gaze data to classify medical images using convolutional neural networks (CNNs). The finding showed a positive result and it shows that gaze data can be used to offer potential medical imaging supervision signals.

### Summary

From the findings, there are a total of 37 studies that related to this study. There are 8 major features used from eye-tracking data for classification, which are pupil size, saccade, fixation, velocity, blink, pupil position, EOG, and gaze point. There are 23 articles from the total used eye features solely from eye-tracking data for classification, while 14 articles with a combination of other signals. From the results, the feature of fixation is the most commonly used (19 studies) by the researchers for classification, followed by pupil diameter from 10 articles. There are 7 studies that used the feature of saccade, 6 studies that used the feature of pupil position, and 4 studies that used the feature of eye blinking. There are 3 studies for features of EOG and gaze point. Finally, the least used feature was velocity, which was only 1 study. Most of the studies used multiple eye features from eye-tracking data instead of a single feature. Furthermore, EEG is the most commonly used combination signal with eye tracking; there are 9 studies that used the combination of EEG, followed by GSR employed in 2 studies. There is 1 study for each use of the following combination of data, which are EIG, face feature, thermal imaging, SSVEP, and fMRI.

## Discussion

This systematic literature review summarizes the articles that use the features from eye-tracking data in classification within 6 years from 2016 to the present. To achieve the objective and the research question of the topic, we reviewed the studies that use eye-tracking technology and identified what eye features are used and extracted from eye data to execute the classification tasks. Overall, we found 37 relevant articles, and they are shown in Tables 1, 2. The articles are categorized into 2 groups: Table 1 shows the study using eye features solely and Table 2 shows the study using the eye features with the combination of other signals. This table shows that the usage of eye features from eye-tracking data has become increasingly accepted to perform classification research.

**Table 2:** Summary of research using eye features with the combination of other signals.

**References**	**Year**	**Topic Domain**	**Objectives**	**Eye Features**	**Other Signals**	**Subjects**	**Eye-trackers**	**Classifier**	**Performance**
Slanzi et al. (2017)	2017	Web users click intention prediction	To propose a behavioral analysis to evaluate the click intention of web users as a mechanism for analyzing web user activities on a website.	Pupil size, gaze positions	EEG	21 (10 males, 11 females)	Sofey eye-tracking system (30Hz)	Logistic Regression	71.09%
Shi et al. (2017)	2017	Emotion recognition	To implement an assessment method for the automated classification of high- and low-quality data using spatial-temporal scan path analysis.	Fixations, scan path	EEG	26 (15 males, 11 females)	SMI eye tracking glasses	Linear SVM	81.7%
Czyzewski et al. (2017)	2017	Real and imaginary motion of limbs classification	To propose an experimental multimodal device with serious brain injuries for the polysensory treatment and stimulation of non-communicative subjects.	Gaze fixation points	EEG	10 (9 males, 1 female)	EyeX Controller	SVM, ANN, Rough sets	91%
Wilbertz et al. (2018)	2018	Decoding of bistable plaid motion perception	To optimize perceptual alternations decoding using the combination of eye and brain signals.	Eye positions	fMRI	20 (8 males, 12 females)	iView XTM MRI (50Hz)	SVM	91%
Guo et al. (2019)	2019	Emotion recognition	To integrate eye image modality into multimodal emotion detection with the combinations of eye movements and EEG.	Pupil diameter, blink	EEG, EIG	16 (6 males, 10 females)	SMI ETG glasses	SVM	79.63%
Jiang et al. (2019)	2019	ASD classification	To investigate atypical visual performance in ASD patients through facial emotion and eye-tracking data.	Eye fixations	Face features	58	Tobii Pro TX300, Tobii X2-60	RF	86%
Thapaliya et al. (2018)	2019	ASD classification	To analyze and evaluate the EEG and eye data for the diagnosis of ASD using a machine learning algorithm.	Fixation times	EEG	52	Tobii X50	SVM, DNN, NB, logistic regression	71–100%
Ding et al. (2019)	2019	MDD classification	To present an approach involving eye-tracking data, EEG, and GSR to identify patients with depression and balanced controls.	Number of fixations, mean glance duration	EEG, GSR	348	Tobii Eye Tracker 4C	SVM, RF, logistic regression	79.63%
Abdelrahman et al. (2019)	2019	Attention classification	To propose a new approach incorporating eye-tracking and thermal imaging to identify attention types.	Fixation duration	Thermal imaging	22 (14 males, 8 females)	Tobii EyeX	SVM, KNN, logistic regression	75–87%
Lin et al. (2019)	2019	Mental spelling classification	To develop a high-speed mental spelling system using eye-tracking and EEG signals.	Gaze position	EEG	5	Tobii Eye Tracker 4C	FBCCA	92.1%
Horng and Lin (2019)	2020	Drowsiness prediction and classification	To design an experiment on physiological cognitive state prediction using multimodal bio-signals.	Gaze point	GSR, brainwave signals, heart rate	10	Tobii Eye Tracker 4C	ANN, SVM	89.1%
Kubacki (2021)	2021	Element sorting	To propose a BCI system for element sorting using SSVEP, EOG, eye-tracking, and force feedback	EOG, eye positions	SSVEP	3	Camera with eyelike library	BCI system	90%
Song et al. (2021)	2021	Vigilance estimation	To propose a DCRA using the combination of EEG and EOG for vigilance estimation	EOG	EEG	23 (11 males, 12 females)	Neuroscan system, eye-tracking glasses	RNN	80–85%
Ha et al. (2021)	2021	Meal-assist detection	To propose a BCI system for meal-assist using triple eye blinking, EEG, and EMG	Eye blink	EEG, EMG	5 males	Computer camera	BCI system	94.67%

There are several major features that can be obtained from eye-tracking data for classification based on the articles found such as pupil size, saccade, fixation, pupil position, and blink. The following features are less to use, which are the velocity of eye rotation, EOG, and gaze point. From the findings, the feature of fixation is the most commonly used by researchers for classification experiments. Fixation is the time span in which the eye is kept aligned with the target for a certain length. This helps to focus the attention of a person to the point of providing visual information. Furthermore, there are some eye features that are rare and they are used and extracted by some researchers to conduct their investigations such as the mean glance duration, dwell time, number of time-adjacent clusters, and spatial density in the eyes. From the findings, most of the studies used multiple eye features instead of a single eye feature in the investigations.

Based on Table 1, most of the studies showed a positive result from the performance. The highest accuracy obtained was 99.3 (Lagodzinski et al., 2018) and 99.21% (Yoo et al., 2019), with the features of EOG and gaze position, respectively. The study of Guo et al. (2021) has the lowest accuracy among the studies found, which achieved an accuracy of 49.32%. Based on Table 2, the least successful approaches achieved 71.09% using pupil size and gaze position with the combination of EEG signals. From the results showed, the usage of eye features with the combination of other signals has a better overall performance compared to the usage of eye features solely. From the findings, EEG is most commonly used as combination signals with eye-tracking data. There are 9 out of 14 articles that used the combination of EEG. EEG is one of the fastest available imaging methods with its high sampling rate. Other types of combination signals included EIG, face feature, thermal imaging, GSR, and heart rate. From the types of classification, there were 5 studies that used eye features on medical classification such as ASD classification, MDD classification, and schizophrenia disorder detection, which have the highest number of studies from the findings followed by 6 articles that use for image classification. From all the papers found, SVM is the most commonly used machine learning classifier in the research of classification using eye features as well as with the combination of other signals. There are 20 out of the total number of studies that used SVM as their classifier in the experiment.

Based on the evidence currently available, eye tracking has a promising research field, with strong implications for the research of classification. Most of the researchers used the major eye features such as fixation and pupil size to conduct their investigations. However, some recent studies utilized minor features from eye-tracking data for their investigation. An exploration of the new feature from eye-tracking data is also a significant task for the improvement of classification study in future perspective. It is an unobtrusive, objective measuring tool that provides accurate and quantitative data. Further research is needed including the application of eye tracking in a wide domain and not only in classification tasks.

## Conclusions

We presented a systematic literature review to investigate what features can be extracted or used from eye-tracking data for classification tasks. In this article, we used four independent electronic databases, which are ScienceDirect, IEEE Xplore, and ACM digital libraries as well as Google Scholar to search the related papers within 6 years (2016 to present). Out of 431 publications initially returned from the search query, there were 37 articles that were directly relevant to our research. There are several features found from eye tracking such as pupil size, saccade, fixations, velocity, blink, pupil position, EOG, and gaze point. Fixation is the most commonly used feature among the studies found. Most of the studies used multiple features from eye-tracking data instead of a single feature. Furthermore, there are also studies that used the combination of other signals for the classification. However, EEG is the most commonly used by the researchers among the studies found in this study. The eye-tracking findings provide substantive information on the responses or actions of the respondent. Several eye features are used for classification, but there was no conclusive evidence as towhich eye feature from eye-tracking data is the most relevant and reliable to classification tasks. It was found that SVM substantially shows a better performance in eye-tracking-based classification compared to other classifiers. In future studies, we plan to perform a review on the usage of features from eye-tracking data across more computational intelligence domains rather than on classification only.

## Author Contributions

JL contributed to the writing of the original draft. JM and JT contributed to the writing, reviewing, and editing as well as supervision and funding acquisition. All authors contributed to the article and approved the submitted version.

## Funding

This study was supported by the Ministry of Science, Technology and Innovation (MOSTI), Malaysia [Grant Number Reference: IF0318M1003 (ICF0001-2018)].

## Conflict of Interest

The authors declare that the research was conducted in the absence of any commercial or financial relationships that could be construed as a potential conflict of interest.

## Publisher's Note

All claims expressed in this article are solely those of the authors and do not necessarily represent those of their affiliated organizations, or those of the publisher, the editors and the reviewers. Any product that may be evaluated in this article, or claim that may be made by its manufacturer, is not guaranteed or endorsed by the publisher.

## Footnotes

^1^ https://ieeexplore.ieee.org/Xplore/home.jsp

^2^ https://dl.acm.org/

^3^ https://www.sciencedirect.com/

^4^ https://scholar.google.com/

^5^ https://www.mendeley.com/?interaction_required=true
